# Epidemiological Society

**Published:** 1897-06-05

**Authors:** 


### Medical Progress and Hospital Clinics.

[*The Editor will be glad to receive offers of co-operation and contributions from members of the profession. All letters should be addressed to* The Editor, at the Office, 28 & 29, Southampton Street, Strand, London, W.C.]

### EPIDEMIOLOGICAL SOCIETY.

A paper was read at the last meeting by Dr. Andrew Davidson on the seasonal fluctuations of epidemic diseases, a subject which, although without doubt of very great interest, must of necessity be treated in a more or less speculative manner. Granting the fact that the various infectious diseases prevail with different intensity at different seasons of the year, each having its own curve in this respect, it was pointed out how very complex were the factors engaged in the production of this seasonal fluctuation. Some epidemic diseases were caused by facultative saprophytes, whose growth in the soil or other surroundings might well be influenced by seasonal changes in meteorological conditions, and others, whose germs were capable of growth and multiplication both within and outside the human body might vary according to the susceptibility of the body, as well as with the varying climatic conditions; while others, such as small-pox and measles, were due to purely obligatory parasites; and it was contended that the seasonal fluctuation in the prevalence of the latter diseases could only be caused by climatic agencies acting on the human body and rendering it more susceptible to contract the infection at one season than another. A very elaborate attempt was made by the author, by comparing the seasonal prevalence of these diseases in different parts of the world, and by eliminating those climatic conditions which did not seem common to all, to arrive at the way in which variations of season produced variations of susceptibility to such diseases, and the conclusion was arrived at that in both cases their prevalence is associated with conditions which produce an increased vulnerability of the respiratory mucous membrane. "The cold increases the susceptibility of the community to the infection. A general survey of the climatic conditions under which the winter and the spring recrudescences of small-pox and measles take place in different parts of the world, and a consideration of the manner in which they are seasonally associated with respiratory diseases point to the same conclusion." The result of the arguments adduced were, then, to the effect that, as the infection of measles and small-pox take place through the air passages, the increased vulnerability determined by the greater prevalence of respiratory diseases in winter and spring furnishes an adequate explanation of the epidemic spread of the former class of maladies at these seasons.

